# Management of medically assisted withdrawal from alcohol in acute adult mental health and specialist addictions in-patient services: UK clinical audit findings

**DOI:** 10.1192/bjo.2023.45

**Published:** 2023-04-11

**Authors:** Julia Sinclair, Thomas R. E. Barnes, Anne Lingford-Hughes, Colin Drummond, Ignatius Loubser, Olivia Rendora, Carol Paton

**Affiliations:** Clinical and Experimental Sciences, Faculty of Medicine, University of Southampton, UK; Division of Psychiatry, Imperial College London, London, UK; and Prescribing Observatory for Mental Health, Centre for Quality Improvement, Royal College of Psychiatrists, London, UK; Division of Psychiatry, Imperial College London, London, UK; National Addiction Centre, Institute of Psychiatry, Psychology and Neuroscience, King's College London, London, UK; Acute and Crisis Directorate, Oxleas NHS Foundation Trust, Dartford, UK; Prescribing Observatory for Mental Health, Centre for Quality Improvement, Royal College of Psychiatrists, London, UK

**Keywords:** Medically assisted alcohol withdrawal, thiamine, Wernicke's encephalopathy, addictions psychiatry, quality improvement

## Abstract

**Background:**

Medically assisted alcohol withdrawal (MAAW) is increasingly undertaken on acute adult psychiatric wards.

**Aims:**

Comparison of the quality of MAAW between acute adult wards and specialist addictions units in mental health services.

**Method:**

Clinical audit conducted by the Prescribing Observatory for Mental Health (POMH). Information on MAAW was collected from clinical records using a bespoke data collection tool.

**Results:**

Forty-five National Health Service (NHS) mental health trusts/healthcare organisations submitted data relating to the treatment of 908 patients undergoing MAAW on an acute adult ward or psychiatric intensive care unit (PICU) and 347 admitted to a specialist NHS addictions unit. MAAW had been overseen by an addiction specialist in 33 (4%) of the patients on an acute adult ward/PICU. A comprehensive alcohol history, measurement of breath alcohol, full screening for Wernicke's encephalopathy, use of parenteral thiamine, prescription of medications for relapse prevention (such as acamprosate) and referral for specialist continuing care of alcohol-related problems following discharge were all more commonly documented when care was provided on a specialist unit or when there was specialist addictions management on an acute ward.

**Conclusions:**

The findings suggest that the quality of care provided for medically assisted withdrawal from alcohol, including the use of evidence-based interventions, is better when clinicians with specialist addictions training are involved. This has implications for future quality improvement in the provision of MAAW in acute adult mental health settings.

Alcohol dependence is a common condition, frequently co-occurring with other mental disorders.^1^ When alcohol consumption is stopped abruptly in a dependent drinker, a physiological withdrawal state develops. There is significant interpersonal variation in the level of alcohol consumption that would result in a person experiencing alcohol withdrawal, but anyone dependent on alcohol is likely to need medically assisted alcohol withdrawal (MAAW).^2^ The aim of MAAW is not only to control the withdrawal symptoms but also to prevent complications such as seizures and delirium tremens, which may cause significant long-term morbidity and mortality. In addition, owing to the increased metabolic load on the brain during alcohol withdrawal, there is a need to mitigate the risk of thiamine deficiency to avoid the development of Wernicke's encephalopathy.^3^ Clinicians should therefore be familiar with the assessment and treatment of alcohol-related disorders relevant to their area of practice. However, little is known about the quality of MAAW when it is conducted on acute adult psychiatric wards and how it compares with care on specialist in-patient alcohol units. We report here on the findings of a clinical audit conducted in the context of a quality improvement (QI) programme that focused on key aspects of MAAW in acute adult mental health wards and specialist alcohol wards.

## Method

The Prescribing Observatory for Mental Health (POMH) is based at the Centre for Quality Improvement at the Royal College of Psychiatrists, London, UK. POMH runs audit-based QI programmes addressing prescribing practice within UK mental health services.^4^ In 2021, as part of a QI programme addressing MAAW on adult psychiatric wards, POMH invited all 66 of its member trusts/healthcare organisations to participate in a clinical audit to benchmark the quality of such practice against evidence-based standards. Those trusts that provided specialist in-patient services for alcohol detoxification were invited to include patients from these units in their audit sample.

The practice standards were derived from two clinical guidelines^5,6^ and a quality standard^7^ addressing the diagnosis, assessment and management of alcohol-use disorders, generated by the National Institute for Health and Care Excellence (NICE), and a consensus statement on the pharmacological management of substance misuse, generated by the British Association for Psychopharmacology.^8^ The practice standards reported on in this paper are as follows:

- practice standard 1: the decision to undertake MAAW for an in-patient should be informed by a documented assessment of drinking history and current daily alcohol intake and a physical examination carried out on admission
- practice standard 2: pharmacotherapy to treat the symptoms of acute alcohol withdrawal should be limited to a benzodiazepine, carbamazepine or clomethiazole
- practice standard 3: thiamine should be prescribed parenterally for in-patients in acute alcohol withdrawal.

In addition, the audit addressed three treatment targets reflecting best practice. These were derived from the same national guidelines as the practice standards, but represented clinical recommendations for which the available evidence fell short of supporting a practice standard, i.e. they would not necessarily apply in all cases. They were included as it was judged that clinicians would be interested in how far their practice was in line with these treatment targets, compared with their peers. The treatment targets were as follows:

- treatment target 1: breath alcohol should be measured as part of the initial assessment for MAAW
- treatment target 2: following MAAW, initiation of relapse prevention medication should be considered
- treatment target 3: after MAAW, referral to specialist alcohol services for continuing management and support should be considered.

All trusts and clinical teams were self-selected in that they chose to participate in the audit, and each trust was invited to include as many clinical teams as it wished. Trusts were asked to submit information that reflected performance against the practice standards for a sample of patients who had been admitted to an acute adult psychiatric ward, psychiatric intensive care unit (PICU) or specialist in-patient addictions unit in the past year and who had required MAAW while an in-patient.

A bespoke standardised data collection tool was used to gather demographic and clinical data from the clinical records of each eligible patient. These included age, gender, ethnicity, the clinical service providing care, status under the Mental Health Act and psychiatric diagnoses. The data collection tool also collected information on the initial clinical assessment of each patient, including documentation of past and current alcohol use, measurement of breath alcohol in the first 24 h after admission and assessment of physical health, including signs of Wernicke's encephalopathy. Regarding alcohol use, questions were included about the ‘number of months of harmful drinking on this occasion’ and the ‘number of units of alcohol consumed each day’ (a UK unit equals 8 g ethanol) prior to admission. If the number of daily units of alcohol was not documented, or the person collecting the data was otherwise unable to provide this figure, details of the daily alcohol consumption were requested in terms of type of alcoholic drink and percentage alcohol and volume, including, if known, the ‘alcohol by volume’ (ABV, abv or alc/vol) measure, to enable the number of units to be calculated. Data were also collected on clinical management, including the medications prescribed for alcohol withdrawal, other medications initiated during alcohol detoxification, the administration of thiamine, the nature of any specialist clinical advice sought during the admission and, at discharge, the care plan in terms of the follow-up clinical service and continuing medication for alcohol dependence. These data were pseudonymous within the trusts but submitted anonymously to POMH. Ethical approval was not required for such an audit-based QI initiative.^9^

### Data analysis

Data were submitted online using Formic clinical audit software, version 5.7.1 for Windows,^10^ and analysed using SPSS for Windows, version 21.0.^11^ Individual participating mental health services were asked to address any data cleaning queries. Descriptive statistics were used to measure performance against the clinical practice standards in the total national audit sample and in three service subsamples: acute adult ward or PICU with no clinical involvement of an addictions specialist, acute adult ward or PICU with input from an addictions specialist, and specialist in-patient addictions unit. For proportions relating to relevant clinical practice variables within these service subsamples, 95% confidence intervals were calculated.

Independent samples *t*-tests were used to compare the means of recorded alcohol use variables across the clinical service subsamples. Binary logistic regression was used to explore whether potential predictive variables (demographic variables and clinical variables, including age, gender, ethnicity, psychiatric diagnoses, diagnosis of alcohol-related liver disease, diagnosis of delirium tremens/Korsakoff's syndrome, number of previous MAAWs, alcohol units consumed a day, the duration of the alcohol use problem, and assessment for Wernicke's encephalopathy) were associated with the use of parenteral thiamine for all patients in the audit sample. For the analysis, the effect of each variable was examined initially using a univariable analysis. Subsequently, the joint effect of explanatory variables was examined in a multivariable analysis, using a backwards selection procedure to retain the statistically significant variables. Where patient characteristics were unknown or not specified, the values were treated as missing.

## Results

Forty-five trusts/healthcare organisations submitted audit data relating to 1255 in-patients who had undergone MAAW, under the care of 173 clinical teams. The demographic and clinical characteristics of the total audit sample are shown in Table 1. Information on previous MAAW had been recorded for 886 (70%) patients, one-third of whom (*n* = 293, 33%) were undergoing MAAW for the first time.

**Table 1:** Demographic and clinical characteristics of the patients in the total national audit sample and three service subsamples: treated on an acute adult psychiatric ward/psychiatric intensive care unit (PICU) with or without specialist input, or in a specialist alcohol unit

			Acute adult wards/PICUs		Specialist alcohol units (*n* = 347)	Total national sample (*n* = 1255)
			No specialist care (*n* = 875)	Specialist care (*n* = 33)		
Gender, *n* (%)	Male		597 (68)	18 (55)	206 (59)	821 (65)
	Female		278 (32)	15 (45)	141 (41)	434 (35)
Age, years	Median age		44	41	46	45
	Range		20–91	20–73	20–80	20–91
	Age bands, *n* (%)	18–30 years	105 (12)	3 (9)	19 (5)	127 (10)
		31–40 years	232 (27)	12 (36)	82 (24)	326 (26)
		41–50 years	246 (28)	14 (42)	115 (33)	375 (30)
		51–60 years	206 (24)	3 (9)	97 (28)	306 (24)
		61+ years	86 (10)	1 (3)	34 (10)	121 (10)
Ethnicity, *n* (%)	White/White British		724 (83)	30 (91)	329 (95)	1083 (86)
	Black/Black British		14 (2)	–	1 (<1)	15 (1)
	Asian/Asian British		28 (3)	2 (6)	5 (1)	35 (3)
	Mixed or other		42 (5)	–	6 (2)	48 (4)
	Not collected/stated or refused		67 (8)	1 (3)	6 (2)	74 (6)
Psychiatric diagnoses (ICD-10), *n* (%)	Mental and behavioural disorders due to use of alcohol (F10)		634 (72)	29 (88)	335 (97)	998 (80)
	Mood disorder, other than bipolar affective disorder (F30–F39)		223 (25)	19 (58)	114 (33)	356 (28)
	Personality disorder (F60–F69)		210 (24)	7 (21)	55 (16)	272 (22)
	Neurotic, stress-related and somatoform disorders (F40–48)		155 (18)	9 (27)	73 (21)	237 (19)
	Schizophrenia spectrum disorder (F20–F29)		178 (20)	1 (3)	7 (2)	186 (15)
	Bipolar affective disorder (F31)		59 (7)	1 (3)	8 (2)	68 (5)
	Other behavioural syndromes associated with physiological disturbances and physical factors (F50–59)		15 (2)	1 (3)	35 (10)	51 (4)
	Attention-deficit hyperactivity disorder (F90)		13 (1)	–	4 (1)	17 (1)
	Disorder of psychological development (F80)		12 (1)	–	5 (1)	17 (1)
	Organic mental disorder (F00–F09)		13 (1)	–	1 (<1)	14 (1)
Alcohol/substance use disorders, *n* (%)	Alcohol-related liver disease (K70.9)		26 (3)	11 (33)	132 (38)	169 (13)
	Mental and behavioural disorders due to multiple drug use and use of other psychoactive substances (F19)		135 (15)	10 (30)	23 (7)	168 (13)
	Mental and behavioural disorders due to use of tobacco (F17)		95 (11)	–	16 (5)	111 (9)
	Mental and behavioural disorders due to use of cannabinoids (F12)		34 (4)	2 (6)	31 (9)	67 (5)
	Mental and behavioural disorders due to use of opioids (F11)		23 (3)	2 (6)	38 (11)	63 (5)
	Mental and behavioural disorders due to use of cocaine (F14)		26 (3)	1 (3)	24 (7)	51 (4)
	Delirium tremens		13 (1)	2 (6)	26 (7)	41 (3)
	Korsakoff's syndrome		9 (1)	–	26 (7)	35 (3)
	Wernicke's encephalopathy (E51.2)		7 (1)	1 (3)	27 (8)	35 (3)
	Benzodiazepine dependence (F13.2)		8 (1)	–	19 (5)	27 (2)
Other medical disorders, *n* (%)	Epilepsy (G40)		13 (1)	1 (3)	3 (1)	17 (1)
	Sleep disorders (G47)		4 (<1)	–	13 (4)	17 (1)
	Traumatic brain injury (S06–S07)		2 (<1)	–	4 (1)	6 (<1)

In total, 889 (71%) patients in the audit sample were on an acute adult ward, 19 (1%) were in-patients on a PICU and 347 (28%) were under the care of a specialist in-patient addictions unit. MAAW had been overseen by an addictions specialist in 33 (4%) of the patients on an acute adult ward, but none of those on a PICU had received such specialist clinical input. The demographic and clinical characteristics of these service subsamples are shown in Table 1. For most of the patients on acute adult wards/PICUs, alcohol withdrawal was unplanned (*n* = 774, 85%), whereas for the majority of patients on specialist alcohol units (*n* = 336, 71%), the admission for MAAW had been planned.

In the total national sample, advice from a physician had been sought for 162 (13%) patients during their in-patient stay, of whom 39 (24%) were transferred to an acute medical bed because of complications of alcohol use or withdrawal.

### Assessment on admission

#### Alcohol history (practice standard 1)

During the initial assessment, information on the daily alcohol intake prior to admission had been recorded in the clinical records of 529 (60%; 95% CI 57–64%) of the 875 patients on an acute adult ward/PICU for whom there had not been specialist input and 28 (85%; 95% CI 73–97%) of the 33 patients where an addictions specialist had been involved in their care. For these 557 patients, the information on their daily alcohol intake had been recorded in terms of units of alcohol in 445 (80%) cases, while in the remaining 112 (20%) the number of units was calculated, during data analysis, from the information provided on the alcoholic drinks they consumed daily. Of the 347 patients treated in a specialist alcohol unit, daily alcohol intake prior to admission was recorded for 343 (99%; 95% CI 98–100%), 315 (92%) of whom had this recorded as units of alcohol whereas for the remaining 28 (8%) the number of units was calculated from the description of the alcoholic drinks consumed daily.

Analysis of the data revealed that the median number of units consumed each day by 343 of the patients admitted to a specialist alcohol unit was 28 (IQR 20–39), compared with 24 (IQR 14–40) for 557 of the patients admitted to an acute adult ward or PICU (*t* = −1.36, d.f. = 898, *P* = 0.175).

There was information on the duration of alcohol use in the records of 212 (24%; 95% CI 21–27%) of the 875 patients on an acute adult ward/PICU without addictions specialist input, 9 (27%; 95% CI 12–42%) of the 33 patients on an acute ward/PICU with specialist input and 239 (69%; 95% CI 64–74%) of the 347 patients treated in a specialist addictions unit. The median duration of the alcohol history for the 239 patients admitted to a specialist addictions unit was 15 years (IQR 5–20), compared with 10 years (IQR 5–20) for the 221 patients on an acute adult ward or PICU (*t* = −2.44, d.f. = 458, *P* = 0.015).

Information on the duration of harmful alcohol consumption immediately prior to admission had been recorded for 213 (24%; 95% CI 21–27%) of the 875 patients on an acute adult ward/PICU for whom there had not been specialist input, 8 (24%; 95% CI 9–39%) of the 33 patients on an acute ward/PICU with specialist input and 189 (54%; 95% CI 49–60%) of the 347 patients treated in a specialist alcohol unit. The median duration of harmful drinking for the 189 patients on a specialist alcohol unit was 12 months (IQR 6–24), compared with 3 months (IQR 1–9.5) for the 221 patients on an acute adult ward or PICU (*t* = −8.66, d.f. = 408, *P* < 0.001).

#### Screening for Wernicke's encephalopathy

Screening for all three signs/symptoms of Wernicke's encephalopathy (disorientation/confusion, ataxia, ophthalmoplegia and/or nystagmus) was recorded for 163 (19%; 95% CI 16–21%) of the 875 patients on an acute adult ward/PICU under non-specialist care and 22 (67%; 95% CI 51–83%) of the 33 patients treated in the same clinical setting but with the involvement of a specialist. Of the 347 patients whose alcohol withdrawal was managed in a specialist unit, 160 (46%: 95% CI 41–51%) had a full assessment of Wernicke's encephalopathy documented in their clinical records. In the total audit sample of 1255 patients, there was no documented assessment of any of the signs or symptoms of Wernicke's encephalopathy for 528 (42%; 95% CI 39–45%).

#### Breath alcohol (treatment target 1)

For the patients on an acute adult ward/PICU, a breath alcohol measurement within 24 h of admission had been documented for 96 (11%; 95% CI 9–13%) of the 875 patients for whom there had been no specialist input and 5 (15%; 95% CI 3–27%) of the 33 patients where an addictions specialist had been involved in their care. Breath alcohol concentration had been measured and documented for 323 (93%; 95% CI 90–96%) of the 347 patients on a specialist unit.

### Management of alcohol withdrawal

#### Medication regimen (practice standard 2)

The medication regimen for MAAW included a benzodiazepine for 874 (96%; 95% CI 95–97%) of the 908 patients on an acute adult ward/PICU and 343 (99%; 95% CI 98–100%) of the 347 on a specialist unit. Of the 908 patients on an acute adult ward/PICU, a fixed-dose reducing regimen with a benzodiazepine was used for 555 (61%; 95% CI 58–64%) and a symptom-triggered medication regimen was used for 256 (28%; 95% CI 25–31%). For the 347 patients on a specialist unit, the respective figures were 179 (52%: 95% CI 46–57%) and 162 (47%; 95% CI 41–52%).

Of the patients on an acute adult ward/PICU, it was documented that the MAAW regimen had been completed as planned for 638 (73%; 95% CI 70–76%) of the 875 patients with no specialist input and 27 (82%; 95% CI 69–95%) of the 33 patients with specialist input. Of the 347 patients treated on a specialist unit, it was recorded that 313 (90%; 95% CI 87–93%) had completed the MAAW regimen.

Of the 908 patients on an acute adult ward/PICU, there was documented initiation of acamprosate during MAAW for 69 (8%; 95% CI 6–10%) of the 875 patients with no specialist involvement and 19 (58%; 95% CI 41–74%) of the 33 patients where there was specialist involvement. Of the 347 patients treated on a specialist unit, such a prescription was recorded for 176 (51%; 95% CI 45–56%). Regarding other medications started during MAAW, of the 875 patients on an acute adult ward/PICU with no specialist involvement in their care, 205 (23%) had prescriptions for antidepressant medication and 222 (25%) had prescriptions for antipsychotic medication. The respective figures for the 33 patients in the same clinical setting but with specialist input were 2 (6%) and 4 (12%). Of the 347 patients treated on a specialist unit, an antidepressant was prescribed for 36 (10%) and an antipsychotic medication for 21 (6%).

#### Use of thiamine (practice standard 3)

The data in Table 2 show the proportion of patients in the audit sample prescribed parenteral and/or oral thiamine. Parenteral (intramuscular or intravenous injection) thiamine was prescribed for 361 (41%; 95% CI 38–44%) of the 875 patients on an acute adult ward/PICU with no specialist involvement and 26 (79%; 95% CI 65–93%) of the 33 patients where there was specialist involvement. Of the 347 patients treated on a specialist unit, a prescription for parenteral thiamine was recorded for 306 (88%; 95% CI 85–91%).

**Table 2:** Prescription of thiamine for patients with and without specialist care for alcohol withdrawal

Prescription of thiamine	Acute adult psychiatric services/PICUs		Specialist alcohol units (*n* = 347)	Total national sample (*n* = 1255)
	No specialist care (*n* = 875)	Specialist care (*n* = 33)		
Parenteral, *n* (%)				
IM followed by oral	282 (32%)	26 (79%)	246 (71%)	554 (44%)
IM only	63 (7%)	–	6 (2%)	69 (5%)
IV followed by oral	9 (1%)	–	43 (12%)	52 (4%)
IV only	7 (1%)	–	11 (3%)	18 (1%)
Oral, *n* (%)				
Oral only	367 (42%)	4 (12%)	38 (11%)	409 (33%)
No documented thiamine prescription, *n* (%)	147 (17%)	3 (9%)	3 (1%)	153 (12%)

PICU, psychiatric intensive care unit; IM, intramuscular injection; IV, intravenous injection.

When potentially relevant clinical variables were examined in the total sample using binary logistic regression, the multivariable analysis found the following to be significantly associated (*P* < 0.01) with the use of parenteral thiamine: evidence of specialist care (i.e. MAAW was conducted under the care of a specialist alcohol unit or overseen by an addictions specialist on an acute adult ward), an ICD-10 F10 diagnostic code (alcohol-related disorders), documented assessment for Wernicke's encephalopathy and the documented presence of delirium tremens and/or Korsakoff's syndrome (Table 3). The odds of being treated with parenteral thiamine were more than seven times higher for those patients receiving specialist care and more than twice as high for those reported to have signs and symptoms of delirium tremens and/or Korsakoff's syndrome.

**Table 3:** Multivariable analysis associated with the documented use of parenteral thiamine

Variable	Category	Odds ratio (95% CI)	*P*
Specialist care	No specialist care	1	<0.001
	Specialist care	7.11 (5.03–10.0)	
ICD-10 F10 diagnosis: alcohol-related disorders	No	1	<0.001
	Yes	1.89 (1.38–2.58)	
Assessment of signs/symptoms of Wernicke's encephalopathy	None	1	0.002
	Partial assessment	1.0 (0.75–1.33)	
	Full assessment	1.72 (1.24–2.39)	
Documented delirium tremens/Korsakoff's syndrome	No	1	0.01
	Yes	2.35 (1.20–4.60)	

### Discharge planning

#### Medication for relapse prevention (treatment target 2)

At the time of discharge, or 4 weeks after completing MAAW if a patient had not been discharged by that time, one or more medications for relapse prevention (acamprosate, naltrexone, disulfiram or baclofen) were prescribed for 117 of the 908 (13%; 95% CI 11–15%) patients on an acute adult ward/PICU, 96 of the 875 (11%; 95% CI 9–13%) patients with no specialist involvement and 21 of the 33 (64%; 95% CI 47–80%) patients for whom there was specialist involvement. Of the 347 patients treated on a specialist unit, such a prescription was recorded for 237 (68%; 95% CI 63–73%). The medication most prescribed for relapse prevention was acamprosate, which was recorded for 84 (10%) of the 875 patients on an acute adult ward/PICU with no specialist involvement and 19 (58%) of the 33 patients where there was specialist involvement. Of the 347 patients treated on a specialist unit, acamprosate was prescribed at discharge for 188 (54%).

#### Referral for continuing management of alcohol use/alcohol-related problems (treatment target 3)

For the 1212 patients in the total audit sample who had been discharged, referral for specialist continuing care for alcohol-related problems following discharge was most commonly to NHS specialist addictions services (*n* = 397, 33%) and community addictions services provided by the third sector (*n* = 361, 30%). Also documented were referrals to another mental health clinical team (*n* = 250, 21%) or dual diagnosis worker/service (*n* = 80, 7%), or referral/signposting to other voluntary support (e.g. Alcoholics Anonymous) (*n* = 224, 18%) or primary care (*n* = 130, 11%). Such referrals were documented in the clinical records of 630 (76%; 95% CI 73–78%) of the 834 patients discharged from an acute adult ward/PICU where there had not been specialist involvement, 31 (94%; 95% CI 86–100%) of the 33 patients discharged from an acute adult ward/PICU where there had been specialist involvement and 337 (98%; 95% CI 96–99%) of the 345 patients discharged from a specialist unit.

## Discussion

The number of alcohol-related hospital admissions has been rising over the past 20 years.^12^ In England, in the year to March 2021, there were approximately 225 000 admissions where the primary or secondary diagnosis was ‘mental and behavioural disorders due to the use of alcohol’ and in approximately 40 000 of these cases this was the primary diagnosis.^13^ The clinical need for MAAW is therefore likely to be increasing. But there has been a marked reduction in the availability of specialist addictions services, including in-patient beds, over the past 10 years and this has put pressure on other parts of the NHS to deliver care for alcohol-use disorder^14^; for example, while the number of MAAW admissions to specialist alcohol units reduced by almost half in the 5 years up to 2019, the number of non-specialist admissions increased to fill this gap.^12^

There is consistency across international guidance for the principles of managing MAAW.^15^ Benzodiazepines are the mainstay of treatment, either in a fixed-dose reducing regimen or prescribed in response to the level of alcohol withdrawal symptoms (symptom triggered). Parenteral thiamine is recommended for the prevention and treatment of Wernicke's encephalopathy. As with all evidence-based interventions, a diagnosis and an assessment of symptom severity are required to effectively target treatment. For individuals admitted to psychiatric in-patient units, who may require unplanned MAAW as part of their treatment, the relevant NICE clinical guidelines and quality standards apply.^5,7^

Our audit data suggest that the quality of assessment and care planning for people who undergo MAAW in UK mental health services may be poorer when an addictions specialist is not involved in their care. Specifically, when MAAW had been conducted on an acute psychiatric ward under the care of a general adult psychiatrist, all of the following were less likely to have been documented compared with a specialist alcohol unit or an acute ward with input from an addictions specialist: a drinking history, an assessment of a breath alcohol level before starting medication for MAAW, an assessment for the signs and symptoms of Wernicke's encephalopathy, a prescription for parenteral thiamine, a prescription for relapse prevention medication and a referral to a relevant community-based service to support relapse prevention.

The NICE-recommended medication for MAAW, a benzodiazepine, was prescribed for nearly all patients, whatever the clinical setting. For the majority of patients, this was in the context of a fixed-dose reducing regimen, with a symptom-triggered medication regimen being less commonly used, particularly for patients on an acute adult ward. This may partly reflect that the NICE guideline *Alcohol-Use Disorders: Diagnosis, Assessment and Management of Harmful Drinking (High-Risk Drinking) and Alcohol Dependence*,^5^ developed by the National Collaborating Centre for Mental Health, recommends that, in an in-patient setting, a symptom-triggered regimen should only be used if staff are ‘competent in monitoring symptoms effectively’ and sufficient resources are available to allow them to do so.

There are few published UK data with which to compare our findings. The impact of addictions specialists has not been systematically studied in acute hospital settings, where published audit data are limited to the positive impact of local alcohol treatment guidelines on the quality of MAAW, particularly the use of parenteral thiamine.^16–18^ One study conducted in a psychiatric hospital almost 20 years ago tested the impact of the introduction of a local protocol/prescription chart on the use of parenteral thiamine during MAAW; although this intervention did increase the use of parenteral thiamine, the involvement of a specialist in a patient's care had the strongest association with the use of parenteral thiamine.^19^

### Do patients who receive MAAW on acute psychiatric wards and specialist alcohol units differ?

As might be expected, there was more psychiatric comorbidity in patients receiving MAAW on acute psychiatric wards, particularly schizophrenia spectrum disorders, compared with those on specialist alcohol units. This may partly explain the greater use of antipsychotic medication for patients on the acute adult wards.

There was no significant difference in the recorded daily alcohol consumption prior to admission between patients treated on an acute ward and those on a specialist unit: the median daily consumption was 24 and 28 units respectively. However, the patients admitted to a specialist unit for planned MAAW had a longer duration of alcohol use and had been drinking at high-risk/dependent levels for much longer immediately prior to admission.

At the time the audit was conducted, there were only five NHS specialist in-patient addictions units in England. Such units are required to offer a supra-regional service; admitting patients from distant services can have an adverse impact on discharge planning. In contrast to the patients undergoing MAAW on acute adult wards, admissions to the specialist units were primarily planned (in practice, this is often after a significant wait) and a primary diagnosis of alcohol dependence was much more common. Nevertheless, our findings suggest that the severity of alcohol dependence in patients admitted to acute mental health services is not significantly different from that seen in specialist units, and so similar skills are required to optimise outcomes.

### Why is breath alcohol rarely measured on acute wards before initiating medication for MAAW?

A measure of breath alcohol is warranted for all patients before starting medication for MAAW. The rationale for this is two-fold: to provide reassurance that breath alcohol is falling prior to administering a benzodiazepine for MAAW (to avoid over-sedation and respiratory depression) and to give an indication of the likely severity of alcohol withdrawal (for example, if a patient is already demonstrating significant symptoms of alcohol withdrawal despite still having high levels of alcohol in the breath/blood, they are likely to be severely dependent).

Although breath alcohol was measured in almost all patients on a specialist unit, it was measured in only one in ten patients on an acute adult ward, irrespective of whether a specialist had been involved in their care or not. This may partly reflect that the vast majority of admissions to specialist units are planned and patients do not stop drinking prior to admission, whereas for patients who are admitted to acute adult wards, MAAW will be an opportunistic but necessary intervention. The former group may have high blood alcohol levels at the point of admission whereas the latter group are perhaps less likely to have high levels, having spent time in an accident and emergency department/triage/an assessment suite prior to arrival on the ward.

Other potential reasons for the low use of breathalysers on acute wards are that these devices, even though they should be considered as part of the essential ward medical equipment, may not be available and/or regularly calibrated and checked. Further, staff may not have received training in the use of breathalysers and may see the use of these devices as having a policing and not medical role, with a positive test being possible grounds for the discharge of a patient for breaking ward rules rather than offering medical support.

### Why might screening for the signs and symptoms of Wernicke's encephalopathy and the prescription of parenteral thiamine in patients receiving MAAW on acute wards not be standard practice?

In contrast to the patients admitted to a specialist unit, for whom the number of units of alcohol consumed daily was documented in the vast majority of cases, this was the case in just less than half of the patients who underwent MAAW on an acute adult psychiatric ward/PICU, despite evidence of the availability of information on the nature and quantity of alcoholic drinks consumed for a greater proportion. These findings suggest a lack of confidence in calculating the number of units consumed and are consistent with the poor knowledge of the alcohol content of commonly consumed alcoholic drinks identified in UK studies of student healthcare professionals and trainee doctors:^20,21^ the majority of respondents underestimated the number of units contained in wine and premium strength beers. Thus, in clinical practice, failure to accurately quantify the daily alcohol intake could lead, in some cases, to an underestimation of current alcohol use and, consequently, an underestimation of the risks of alcohol withdrawal, including the development of Wernicke's encephalopathy.

Documented assessment of the signs of Wernicke's encephalopathy and the prescription of parenteral thiamine were both significantly more common for patients whose MAAW was overseen by an addictions specialist. As Wernicke's encephalopathy is a potentially devastating complication of MAAW in severely dependent drinkers, with significant future costs in terms of health and care services due to permanent brain damage, it is core medical competence to consider the risk of developing it, to identify that it is present and then treat it appropriately.^22,23^ Given the apparently similar risk of Wernicke's encephalopathy in patients with and without specialist input (based on the levels of alcohol consumption prior to admission), it is a source of concern that there was no recorded assessment of any of the signs and symptoms of Wernicke's encephalopathy in over 40% of the total patient sample and that the patients with no such documented assessment were less likely to be prescribed parenteral thiamine. Besides the factors described above that may at least partly explain the poor documentation of drinking history in patients who underwent MAAW in an acute psychiatric ward, a further potential explanation for our findings is that MAAW is rarely the primary reason for admission to such a ward and the immediate care plan for a patient prioritises the management of behaviours that challenge and symptoms of known or suspected psychiatric illness. Most mental health trusts will have a clinical policy/guideline for managing MAAW but it may be that without specialist addictions input into teaching and training at trust level there is a lack of awareness of the importance of appropriate management of alcohol withdrawal to prevent poor patient outcomes, so local policies/protocols are poorly implemented.

### Why might patients who received MAAW on acute psychiatric wards not be referred on to appropriate community-based alcohol services or prescribed relapse prevention medication?

Clinical guidelines^5,7,8^ clearly recommend that medication for relapse prevention should be offered after MAAW as part of a wider psychosocial treatment plan. In contrast to these recommendations, the audit data revealed a relatively low use of relapse prevention medications in patients with no specialist input. This may partly reflect that patients who had an unplanned detoxification from alcohol during admission to an acute adult ward were not yet ready to commit to abstinence or accept a referral to a specialist service. For such patients, motivational interviewing could be an essential part of their wider care package during admission and on discharge. Our finding may also partly reflect that relapse prevention medicines are often categorised by Area Prescribing Committees as ‘hospital only’ or ‘requiring shared care’. If so, this presents structural barriers inhibiting the use of relapse prevention medications for the in-patient clinical team, as it cannot support a community case-load: post-discharge, a patient may have intermittent, if any, contact with a community mental health team (many of which have no system to support routine repeat prescribing). Specialist community addictions services are commissioned and provided separately, and a general practitioner requires a specialist to be identified with whom care can be shared. Overcoming these barriers requires a system solution, although this is rarely seen as a priority in local health economies and may therefore remain elusive. Further impediments are the lack of addictions specialists to advocate for practicable solutions to remove these barriers to good patient care and the stigma associated with alcohol dependence.

### What can be done to improve the quality of MAAW in mental health settings?

The Royal College of Psychiatrists’ 2022 curriculum for general psychiatry^24^ requires all core trainees in psychiatry to ‘Demonstrate skills in assessing and managing patients with addictions’. This stipulation could provide an opportunity to upskill the medical workforce in this area and improve MAAW for patients admitted to acute psychiatric in-patient units. There are emerging regional ‘addictions tutor networks’ (overseen by the Addictions Specialty Advisory Committee within the Royal College of Psychiatrists), which will facilitate the assessment of these new competencies and would be well placed to encourage further QI work in this area. This is especially important as alcohol dependence is a common comorbidity in people with severe mental illness, and yet these individuals face numerous attitudinal as well as structural barriers that prevent them from receiving optimised person-centred care.^25^

In summary, the findings of this clinical audit suggest that the severity of alcohol dependence in those patients undergoing MAAW is similar across adult mental health and specialist alcohol services, but there is less use of evidence-based interventions in the former; notably, the use of parenteral thiamine to protect against the development of Wernicke’s encephalopathy and use of medication aimed at reducing relapse into problem drinking after MAAW. Planned competency-based training in addictions for psychiatric trainees is a positive development that could improve the quality of MAAW in non-specialist wards but more needs to be done to enable the development of care pathways that support community-based prescribing for relapse prevention.

### Strengths and limitations

- Given the relatively large sample size and the submission of data by the majority of mental health trusts, the findings presented in this paper are likely to be representative of current practice regarding MAAW in in-patient adult mental health services in the UK. However, they may not be generalisable outside of this clinical setting.
- We cannot confirm the methods used by trusts to identify their audit samples. However, given the number of participating services, systematic bias would seem unlikely.
- All the audit data were systematically collected over the same time period, using a standard data collection tool.
- The subsamples of patients undergoing MAAW on an acute adult ward or in a specialist service were large enough to allow for clinically valid comparisons.
- With respect to performance against the practice standards and treatment targets, the audit data were drawn primarily from documentation in the clinical records and therefore some of the findings are dependent on the quality of record keeping.

## Data Availability

The aggregated data-set that supports these findings is not openly available. Membership agreements between POMH and participating mental health services state that each mental health service owns its own data-set and that this will not be shared by POMH with any third party. POMH is restricted to reporting on analyses based on the aggregated national data-set.
